# Subregion-based radiomics analysis for predicting the histological grade of clear cell renal cell carcinoma

**DOI:** 10.3389/fonc.2025.1554830

**Published:** 2025-05-27

**Authors:** Xue Lv, Xiao-Mao Dai, Dai-Quan Zhou, Na Yu, Yu-Qin Hong, Qiao Liu

**Affiliations:** Department of Radiology, The Third Affiliated Hospital of Chongqing Medical University, Chongqing, China

**Keywords:** clear cell renal cell carcinoma, histological grade, radiomics, machine learning, molecular biological mechanisms

## Abstract

**Purpose:**

We explored the feasibility of constructing machine learning (ML) models based on subregion radiomics features (RFs) to predict the histological grade of clear cell renal cell carcinoma (ccRCC) and explore the molecular biological mechanisms associated with RFs.

**Methods:**

Data from 186 ccRCC patients from The Cancer Imaging Archive (TCIA) and 65 ccRCC patients from a local hospital were collected. RFs were extracted from entire tumor regions and subregions, which were segmented via a Gaussian mixture model (GMM). ML models and radiomics scores (radscores) were developed on the basis of candidate RFs. A RFs-related gene module was identified. Key signaling pathways were enriched, and hub genes were identified.

**Results:**

Two subregions were segmented. The logistic regression (LR) and support vector machine (SVM) models constructed using 3 candidate RFs selected from subregion 1 demonstrated the best predictive performance, with AUCs of 0.78 and 0.77 for the internal test set and 0.74 and 0.77 for the external validation set, respectively. Radscores stratified ccRCC patients into high- and low-risk groups, with high-risk individuals exhibiting poorer overall survival (OS) for the internal test set. Radiogenomic analysis revealed that RFs were associated with signaling pathways related to cell migration, cell adhesion, and signal transduction. The hub genes CTNNB1 and KDR were identified as being associated with RFs.

**Conclusion:**

We revealed an association between RFs and tumor biological processes. The proposed subregional radiomics models demonstrated potential for predicting the histological grade of ccRCC, which may provide a novel noninvasive predictive tool for clinical use.

## Introduction

Renal cell carcinoma (RCC) is one of the most common urological tumors worldwide, with clear cell renal cell carcinoma (ccRCC) representing the most prevalent subtype. This subtype accounts for approximately 70%-85% of RCC cases (1, 2). The World Health Organization/International Society of Urological Pathology (WHO/ISUP) have classified ccRCC into four grades on the basis of the prominence of nucleoli, with higher grades having poorer prognoses than lower grades do (3–5). Early and accurate grading is crucial for determining therapeutic strategies and predicting patient outcomes. In clinical practice, the preoperative pathological grade relies mainly on invasive tissue biopsy, which may present some risks and complications, such as bleeding, local infection and tumor seeding along the needle tract (6). Noninvasive imaging techniques such as ultrasound, CT, and MRI have been widely used, but they lack clear quantitative standards. Therefore, the development of a new noninvasive, quantitative method for predicting pathological tumor grade is highly clinically important.

Radiomics, an emerging field in medical imaging, involves the extraction of high-throughput information from medical images, offering a novel avenue for the noninvasive characterization of tumor phenotypes (7–10). Some studies have confirmed the role of radiomics in predicting the tumor-node-metastasis (TNM) stage, pathological grade, distant metastasis, and prognosis of ccRCC (11–14). However, to our knowledge, ccRCC quantitative feature analysis has focused on the entire tumor region without considering intratumoral variation in different subregions. To better capture the intratumoral heterogeneity of ccRCC, this study employs an innovative approach by segmenting tumors into multiple distinct subregions and extracting RFs from each subregion for model construction. This subregional radiomics analysis strategy provides richer biological information than traditional methods do, which may enhance the accuracy of ccRCC pathological grade prediction.

Despite its potential for enhancing predictive and prognostic accuracy, the poor interpretability of RFs has significantly hindered their clinical application. Therefore, this study explores the associations between RFs and potential biological mechanisms and the biological significance underlying RFs. This study contributes to revealing the molecular mechanisms underlying these features, thereby enhancing their practicality in clinical decision-making.

This study aimed to extract RFs from each subregion to construct machine learning (ML) models for predicting the pathological grade of ccRCC and explore the underlying gene expression patterns and key biological pathways associated with RFs. We also compared the prediction accuracy of subregional models with that of the entire regional model.

## Materials and methods

### Study design

**Figure 1:**
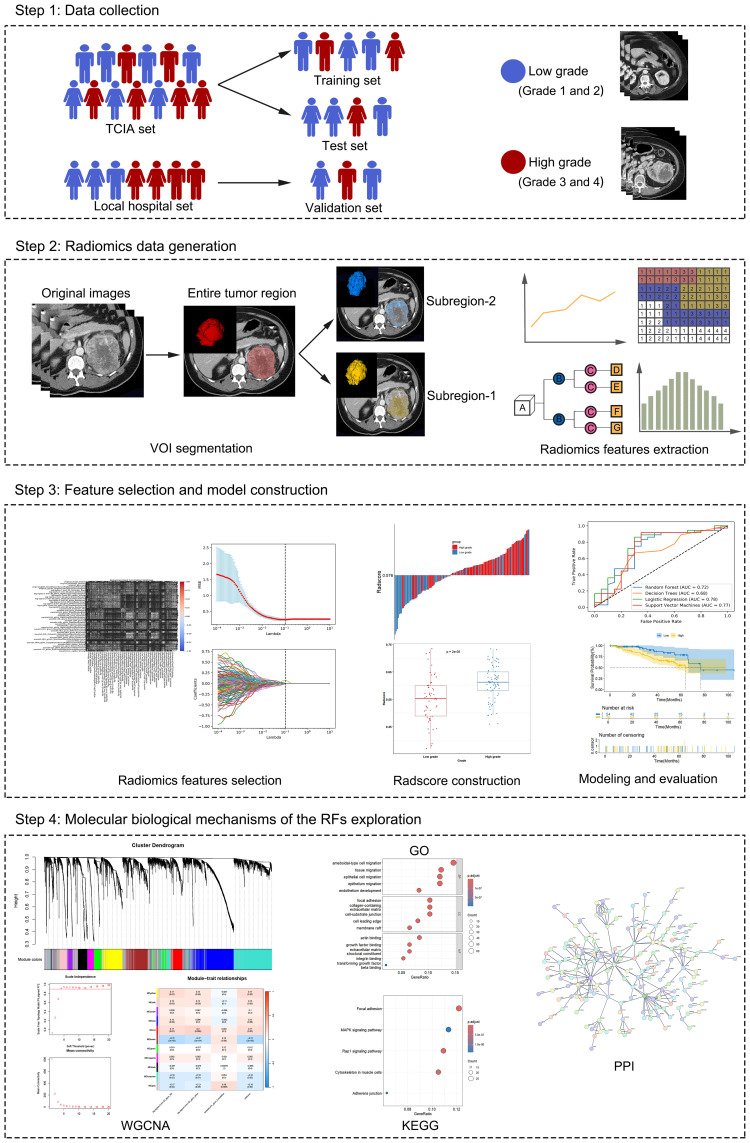
Workflow of the study.

**Figure 1** depicts the schema of the present study, which includes the following steps: (1) Data collection: Data from ccRCC patients were collected from two institutions. These data include CT images, clinical characteristics, and transcriptomic data. (2) radiomics data generation: The volume of interest (VOI) was delineated on the parenchymal phase contrast-enhanced CT (CECT) images, followed by subregion clustering and RF extraction from each region. (3) Feature selection and model construction: Based on the candidate RFs selected via Spearman correlation analysis and least absolute shrinkage and selection operator (LASSO) regression analysis, ML models and the radscore for predicting the pathological grade of ccRCC were constructed and validated. (4) Molecular biological mechanisms of RF exploration: Genes associated with RFs were identified via weighted gene coexpression network analysis (WGCNA). These genes were subsequently subjected to gene ontology (GO) and Kyoto Encyclopedia of Genes and Genomes (KEGG) pathway enrichment analyses to explore their biological pathways. Additionally, a protein–protein interaction (PPI) network was employed to identify the hub genes.

### Patients

This multi-institutional retrospective study involved a total of 186 patients with ccRCC in the discovery cohort. These patients were randomly divided into a training set and an internal test set at a 7:3 ratio. Parenchymal phase CECT images were collected from The Cancer Imaging Archive (TCIA) database, and transcriptomic data and relevant clinical information were extracted from The Cancer Genome Atlas (TCGA). Institutional review board approval was waived since patient data in TCIA and TCGA were deidentified. A total of 64 patients from the local hospital between July 2016 and December 2023 were used as an external validation set. The histological grade was classified into a low-grade group (grades 1 and 2) and a high-grade group (grades 3 and 4).

**Figure 2:**
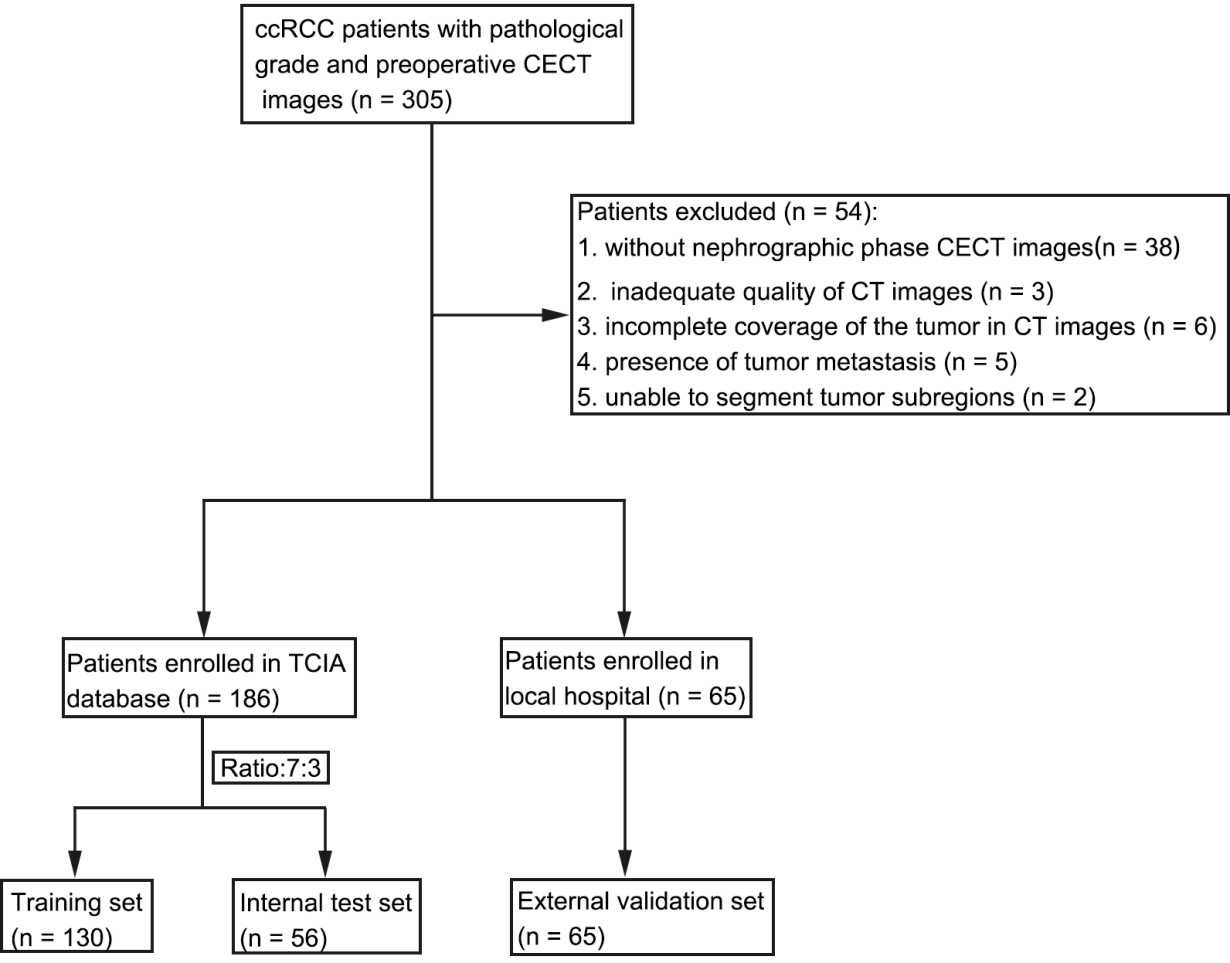
Flowchart of patient selection.

The inclusion criteria for all patients were as follows: (1) confirmation of ccRCC with pathological grading on the basis of postoperative pathology; (2) complete preoperative CECT images. The exclusion criteria were as follows: (1) no parenchymal phase CECT images; (2) inadequate quality of CT images; (3) incomplete coverage of the tumor in CECT images; (4) presence of tumor metastasis; and (5) inability to segment tumor subregions (**Figure 2**).

### Imaging protocol

CT images from the local hospital were acquired using a multi-slice spiral CT scanners (Revolution CT; GEhealthcare). All patients were administered 400 ml of water orally 20 min before the examination. For the enhanced scan, a high-pressure syringe was used to inject a nonionic contrast agent (80–90 mL iohexol 350 mgI/mL, Hengrui Medicine Co., Ltd.) at a dose of 3 mL/s. After 60–70 s, diaphragm-to-kidney images of the parenchymal phase were obtained. The image parameters were as follows: matrix, 512 × 512; rotation time, 0.5 s; tube voltage, 120 kV; tube current, 200–600 mA; detector coverage, 80 mm; pitch, 0.992:1; slice thickness, 2.5 mm; and reconstruction interval, 2.5 mm.

### VOI delineation and subregion clustering

VOIs were manually delineated by a radiologist (X.L. with 7 years of experience in CT interpretation) via the open-source software 3D slicer software (version 5.6.2) and were agreed upon by another radiologist (X.M.D. with 20 years of experience in CT interpretation). The two radiologists were blinded to the patients’ pathological grade. Intraclass correlation coefficients (ICCs) were used to assess the reproducibility of the RFs. Twenty VOIs were delineated again by two radiologists (X.L. and X.M.D.) one month later. The RFs with both intra- and interobserver ICCs greater than 0.75 were retained. For additional details on the ICC analysis, please refer to **Supplementary Material S1**.

A Gaussian mixture model (GMM) was used for clustering subregions with similar RFs. The optimal number of clusters was determined by the Bayesian information criterion (BIC). The number of clusters from 2 to 10 was tested in this study (15, 16).

### Feature extraction

Because the images originated from various CT scanners with different imaging parameters, it was necessary to preprocess the images to standardize the data analysis. The voxel sizes of all the CT images were reconstructed to 1×1×1 mm3, and 25 grey levels were used to discretize their intensities. A total of 1130 RFs were extracted from each region via the PyRadiomics platform (version 3.0.1) in Python software (Python Software Foundation, version 3.7.6) (17). The extracted RFs were resampled and normalized linearly in the range of 0 to 1. The RFs used in this study are described in **Supplementary Material S2**.

### Feature selection

To reduce multicollinearity among features, we conducted a Spearman correlation analysis, retaining only one of the two features when r > 0.9. Then, LASSO regression analysis with 5-fold cross-validation was performed to select the candidate RFs for each region. Afterwards, the radiomics scores (radscores) were calculated through a linear combination of the selected features weighted by their respective coefficients. The Youden index was used to select the best cut-off value where the sum of sensitivity and specificity was maximized. Patients were stratified into high-risk and low-risk groups on the basis of the optimal cut-off value.

### Model construction and evaluation

Candidate RFs selected from each region were used to construct four ML models, including random forest (RF), logistic regression (LR), decision tree (DT), and support vector machine (SVM). These models were trained to predict the histological grade of ccRCC. The predictive performance of the models was evaluated by plotting the receiver operating characteristic (ROC) curve and calculating the corresponding area under the curve (AUC) for the internal test set and the external validation set. The accuracy, precision, recall and F1 score of the models were also calculated.

### Gene coexpression module construction

We utilized WGCNA (13) to explore the gene expression patterns associated with RFs. Initially, we calculated the Pearson correlation coefficients between genes and determined appropriate weights via the soft-thresholding method to construct a scale-free network. We then applied the topological overlap measure (TOM) to refine the network and eliminate spurious correlations. The highly coexpressed genes were subsequently clustered into modules through hierarchical clustering analysis. We assessed the correlation between these modules and the RFs, identifying the most significantly related gene module. To ensure biological relevance, we set a minimum threshold of 100 genes per module.

### Functional enrichment analysis

To investigate the biological significance of the gene module most related to RFs, the GO database was used to evaluate biological processes (BP), cellular components (CC) and molecular functions (MF). The KEGG database was used to identify key signaling pathways. The top five results in ascending order of P value (*p* < 0.05) are displayed in this study. The PPI network within the gene module was constructed with Cytoscape software (version 3.10.0) via the online STRING database. The two most connected genes in the PPI network were identified as hub genes.

### Statistical analysis

Statistical analysis was performed with R software (version 3.6.0) and Python (version 3.6.8). Continuous variables are expressed as the means ± standard deviations (SDs) (for normally distributed features) or the medians and interquartile ranges (for nonnormally distributed features). Categorical variables are expressed as counts with percentages. Categorical variables were compared via chi-square tests or Fisher’s exact tests, whereas independent samples t tests or Mann–Whitney U tests were used to compare continuous variables. Correlation analysis was performed via Pearson or Spearman correlation analysis. The association of the radscore with overall survival (OS) was evaluated via Kaplan–Meier (KM) analysis and compared via the log rank test. P values less than 0.05 were considered statistically significant.

## Results

### Clinical characteristics

The clinical characteristics of the patients in the training set, internal test set and external validation set are shown in **Table 1**. There were no statistically significant differences in age, sex or laterality between the low- and high-grade groups (*p* >  0.05). The TNM stage significantly differed in the training set, with the low-grade group demonstrating a lower TNM stage than the high-grade group did (*p*  <  0.05). However, these differences were not statistically significant in the internal test set.

**Table 1:** Clinical information of this study.

Feature	Training set (n=130)		*p*	Internal test set (n=56)		*p*	External validation set(n=65)		*p*
	High-grade group (n=76)	Low-grade group (n=54)		High-grade group (n=36)	Low-grade group (n=20)		High-grade group (n=12)	Low-grade group (n=53)	
Age, (years)	58.70 ± 12.68	58.24 ± 11.22	0.83	61.03 ± 10.72	63.65 ± 12.97	0.42	62.67 ± 10.52	59.89 ± 11.73	0.45
Sex, n (%)			0.13			0.20			0.54
Male	57 (75.00%)	33 (61.11%)		24 (66.67%)	9 (45.00%)		9 (75.00%)	32 (60.38%)	
Female	19 (25.00%)	11 (38.89%)		12 (33.33%)	11 (55.00%)		3 (25.00%)	21 (39.62%)	
Laterality, n (%)			0.99			0.32			1
Right	41 (53.95%)	30 (55.56%)		19 (52.78%)	7 (35.00%)		6 (50.00%)	25 (47.17%)	
Left	35 (46.05%)	24 (44.44%)		17 (47.22%)	13 (65.00%)		6 (50.00%)	28 (52.83%)	
TNM stage, n (%)			<0.001^*^			0.06			/
High stage(3/4)	41 (53.95%)	9 (16.67%)		18 (50.00%)	4 (20.00%)		/	/	
Low stage(1/2)	35 (46.05%)	45 (83.33%)		18 (50.00%)	16 (80.00%)		/	/	

^*^Statistically significant difference.

### Subregion clustering and feature selection

**Figure 3:**
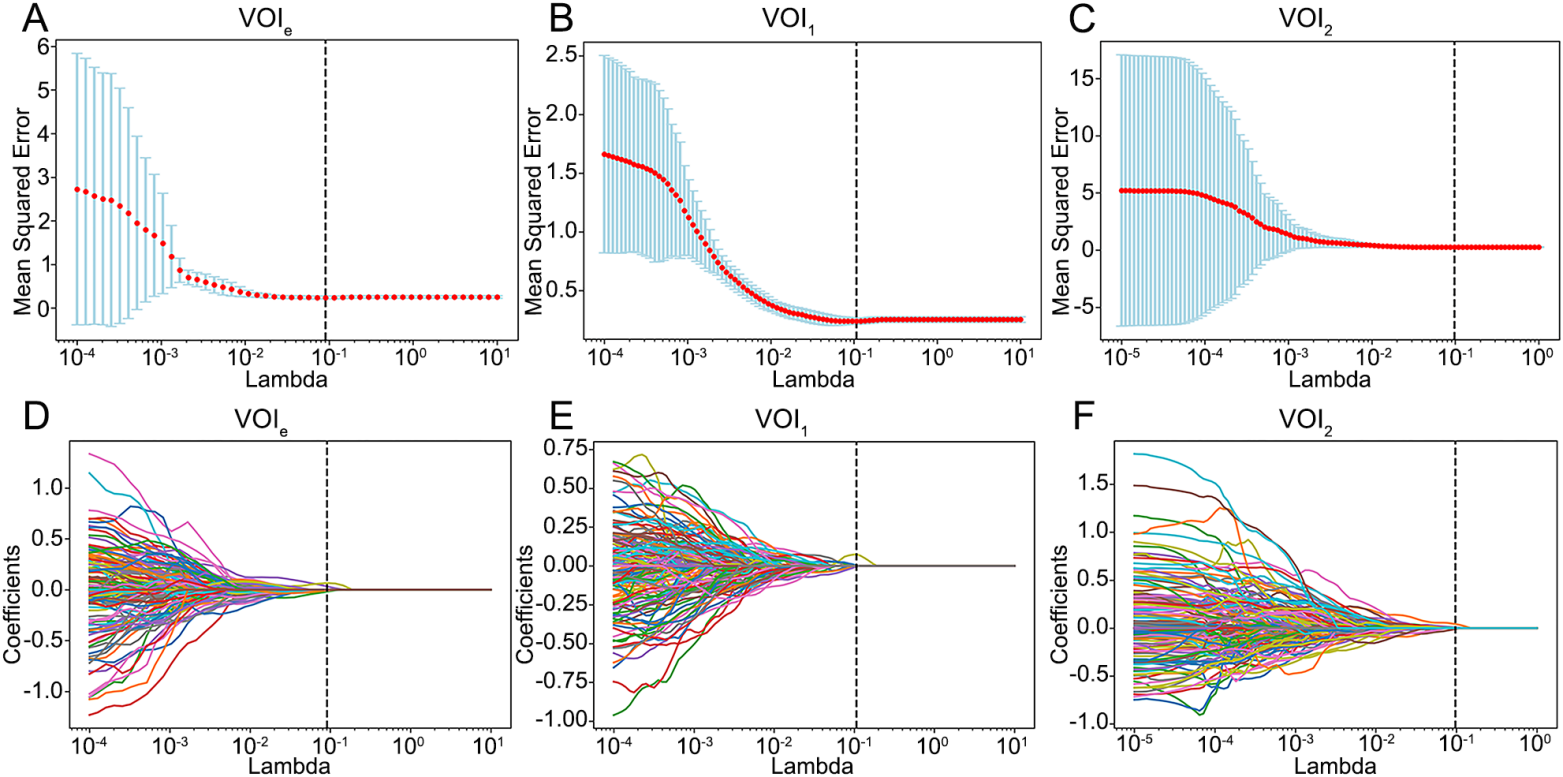
Feature selection using the LASSO model. **(A–C)** Selection of the tuning parameter lambda using 5-fold cross-validation and minimum criterion. The optimal lambda was determined as the value that minimizes the mean-squared error. Vertical dotted lines were drawn at the optimal lambda. **(D–F)** The trajectory of coefficients for each RF in the LASSO model across varying lambda. The vertical dotted line showed the optimal lambda. VOIe, entire region, VOI1, subregion 1, VOI2, subregion 2.

The optimal number of clusters was determined to be 2 via the GMM (**Supplementary Figure S1**); thus, the entire region (designated VOI_e_) was clustered into 2 subregions (designated VOI_1_ and VOI_2_). A total of 1130 RFs were extracted from VOI_e_, VOI_1_ and VOI_2,_ among which 903 reproducible RFs were retained in the downstream analysis (ICCs > 0.75). Following feature selection via Spearman correlation and LASSO regression analysis (**Figure 3**), 5, 3, and 4 candidate RFs were selected for model development from VOI_e_, VOI_1_, and VOI_2_, respectively.

### Model performance and radscore construction

**Figure 4:**
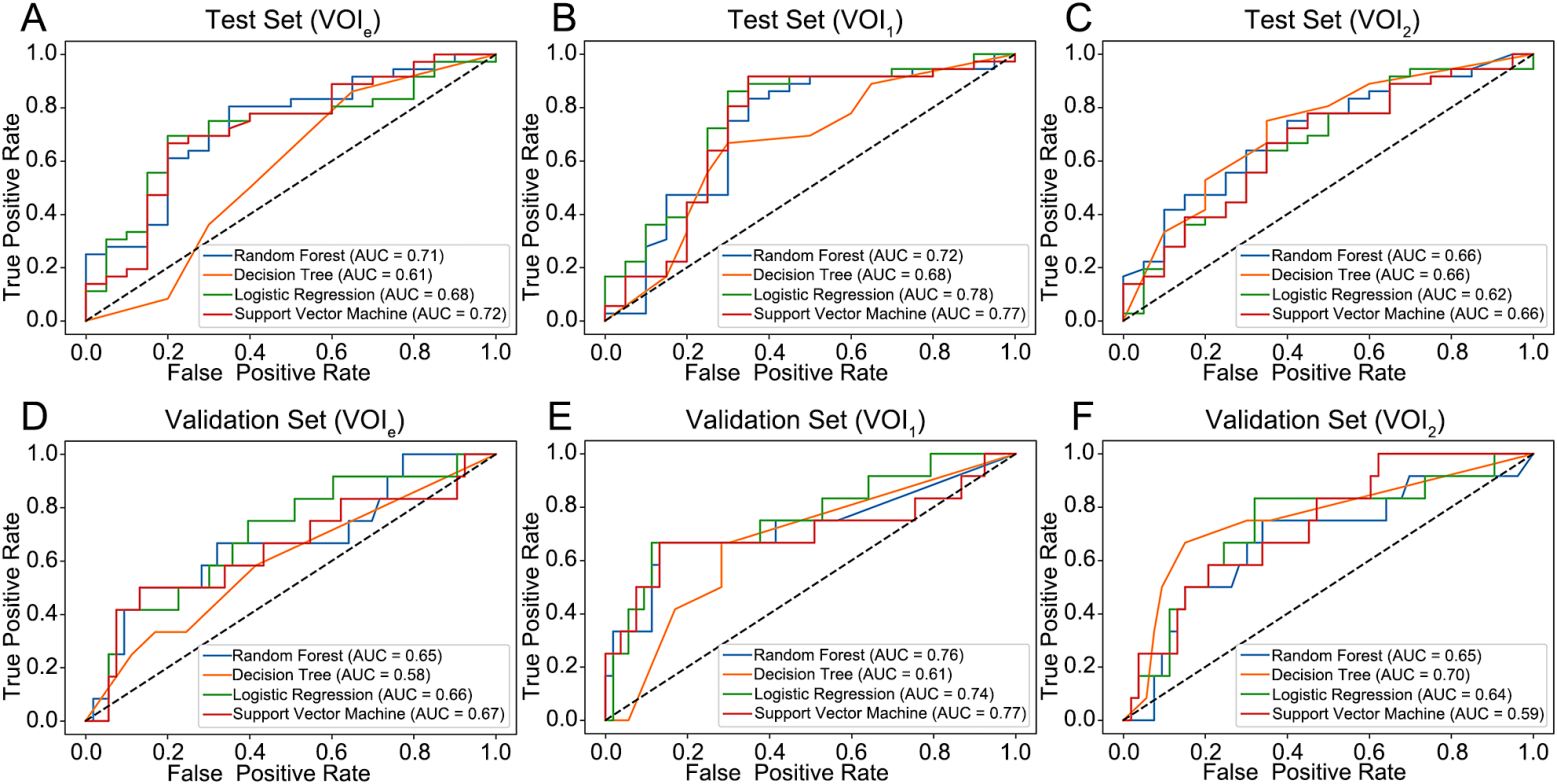
Performance of the four models for predicting the histological grade of ccRCC. **(A–C)** ROC curve for the internal test set. **(D–F)** ROC curve for the external validation set. VOIe, entire region, VOI_1_, subregion 1, VOI_2_, subregion 2.

Compared with those based on VOI_2_, the models constructed on VOI_1_ demonstrated superior predictive performance (**Figure 4**; **Table 2**). Among these models, the LR and SVM models performed better, with AUCs of 0.78 and 0.77 for the internal test set and 0.74 and 0.77 for the external validation set, respectively. The radscore was subsequently calculated by summing the 3 candidate RFs from VOI_1_ and multiplying them by their corresponding coefficients in the LASSO model. The formula used to calculate the radscore is shown in **Supplementary Material S3**.

**Table 2:** Prediction performance of models.

Model	Internal test set					External validation set				
	Accuracy	Precision	Recall	F1-score	AUC	Accuracy	Precision	Recall	F1-score	AUC
VOI_e_										
RF	0.73	0.74	0.73	0.73	0.71	0.74	0.78	0.74	0.75	0.65
LR	0.70	0.70	0.70	0.70	0.68	0.82	0.80	0.82	0.81	0.66
DT	0.68	0.66	0.68	0.65	0.61	0.58	0.75	0.58	0.63	0.58
SVM	0.71	0.74	0.71	0.72	0.72	0.83	0.82	0.83	0.82	0.67
VOI_1_										
RF	0.73	0.74	0.73	0.74	0.72	0.82	0.84	0.82	0.82	0.76
LR	0.80	0.80	0.80	0.80	0.78	0.83	0.84	0.83	0.83	0.74
DT	0.68	0.71	0.68	0.68	0.68	0.68	0.76	0.68	0.71	0.61
SVM	0.80	0.80	0.80	0.80	0.77	0.83	0.85	0.83	0.84	0.77
VOI_2_										
RF	0.70	0.69	0.70	0.69	0.66	0.74	0.78	0.74	0.75	0.65
LR	0.64	0.65	0.64	0.65	0.62	0.78	0.78	0.78	0.78	0.64
DT	0.66	0.68	0.66	0.67	0.66	0.83	0.83	0.83	0.83	0.70
SVM	0.66	0.68	0.66	0.67	0.66	0.80	0.77	0.80	0.78	0.59

AUC, area under the curve; RF, random forest; LR, logistic regression; DT, decision tree; SVM, support vector machine.

### Differences in the radscore among different clinical groups

**Figure 5:**
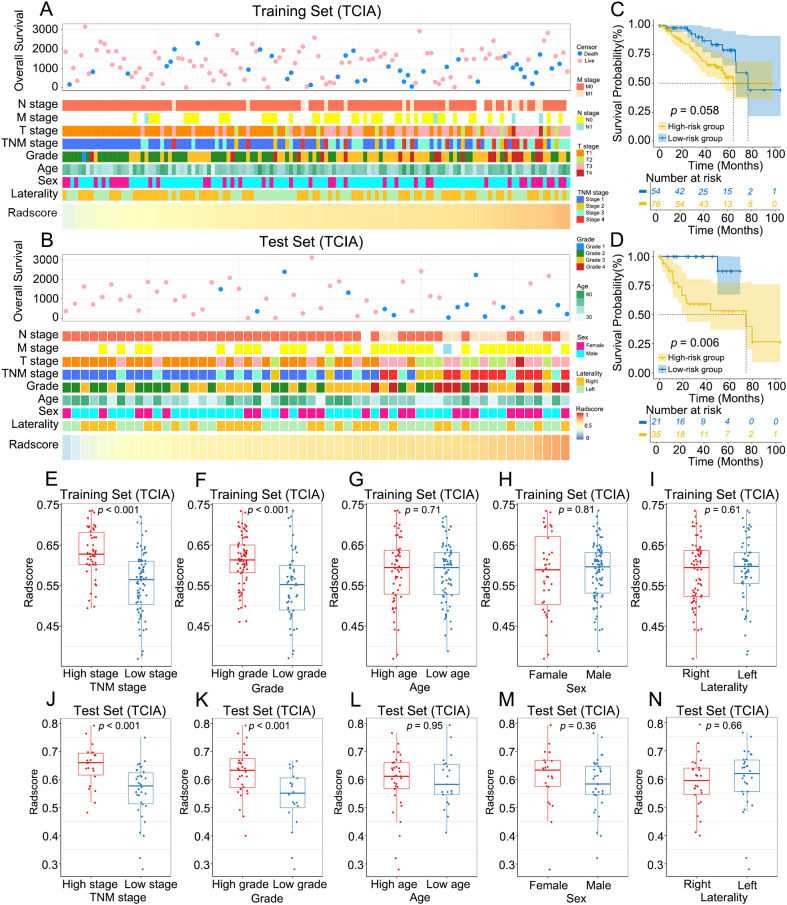
Association between radscore and clinical characteristics of ccRCC. **(A, B**) The landscape of radscore-related clinical characteristics of ccRCC in the training set and the internal test set. **(C, D)** KM curves of OS for low-risk and high-risk groups in the training set and the internal test set. **(E–N)** Differences in radscore between different clinical subgroups in the training set and the internal test set.

The relationships between the radscore and clinical characteristics are shown in the heatmaps (**Figures 5A, B**; **Supplementary Figure S2A**). Patients with varying radscores presented distinct patterns of clinical characteristics. Increases in radscore, TNM stage and histological grade led to asymmetric distributions. In the training set, the radscore was significantly greater in the higher-grade and higher-stage groups. These findings were validated in both the internal test set and external validation set (**Figures 5E–N**; **Supplementary Figure S2B–E**). There was no significant difference in the radscore among the age, sex, and laterality subgroups.

The optimal radscore cut-off value was 0.576, and patients were categorized into a high-risk group (radscore ≥ 0.576) and a low-risk group (radscore < 0.576) (**Supplementary Figure S3**). KM curves demonstrated that OS was significantly lower in the high-risk group than in the low-risk group in the internal test set (*p* = 0.006), whereas no significant difference in OS was observed between the two groups in the training set (*p* = 0.058) (**Figures 5C, D**).

### Identification of the RFs-related gene module

**Figure 6:**
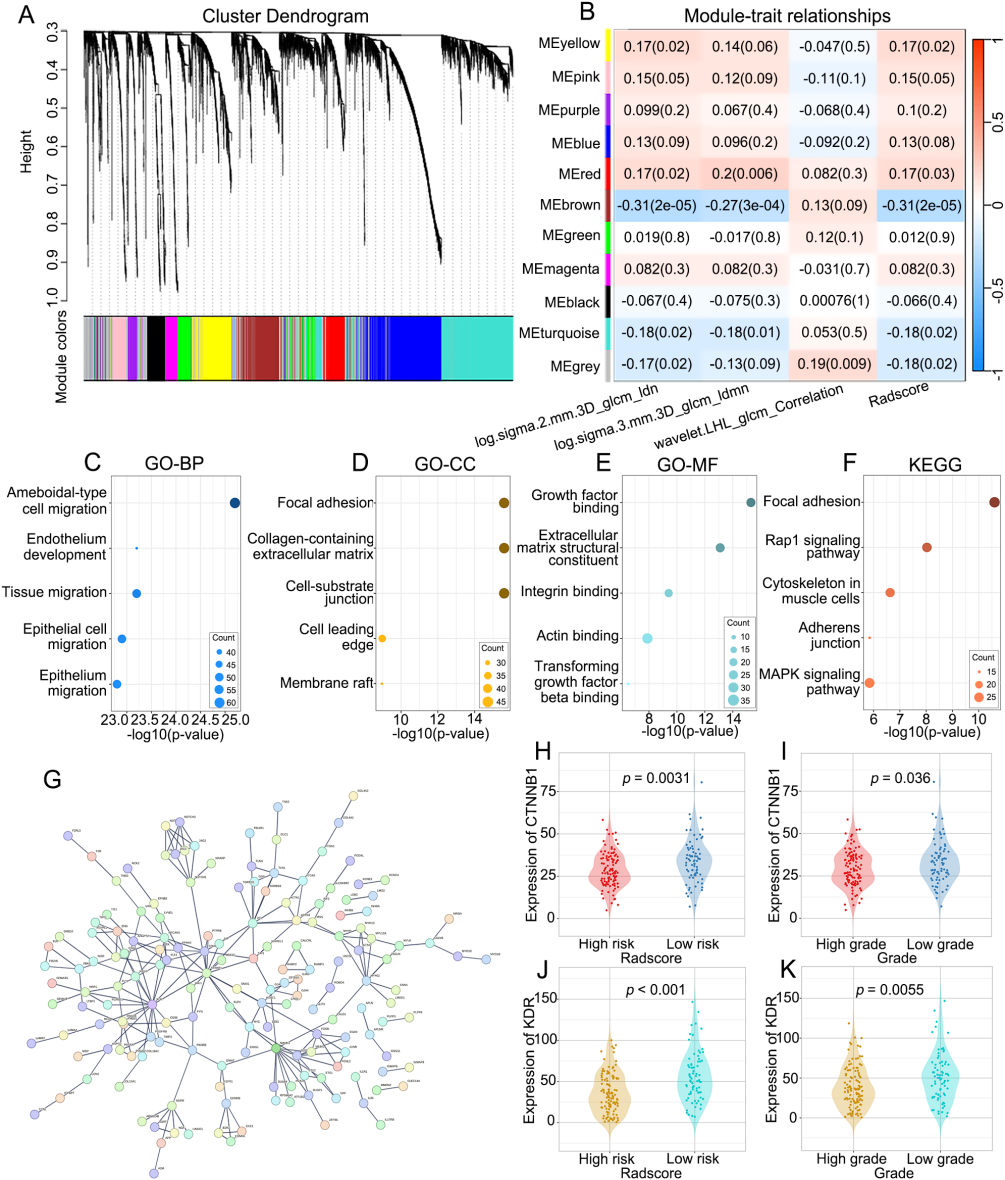
The molecular biological mechanisms of the RFs. **(A)** The cluster dendrogram of genes. Each branch represents one gene and each color below denotes one co-expression gene module. **(B)** Heatmap of the correlation analysis between co-expression gene module and RFs. The MEbrown module displayed the most significant correlation. **(C–E)** GO enrichment analysis of genes in the MEbrown module, including biological processes (BP), cellular components (CC) and molecular functions (MF) categories. **(F)** KEGG pathway analysis of genes in the MEbrown module. **(G)** PPI network indicating the interactions among the genes in the MEbrown module. **(H, J)** Differences in the expression of CTNNB1 and KDR between low-risk and high risk-groups. **(I, K)** Differences in the expression of CTNNB1 and KDR between high-grade and low-grade groups.

The results of WGCNA are summarized in **Figures 6A, B**. We selected β = 5 as a suitable soft threshold for constructing the scale-free network. Eleven coexpressed gene modules were derived via hierarchical clustering analysis, among which the MEbrown module displayed the most significant correlation with radiomics (Pearson correlation r = -0.31, *p* < 0.001). Consequently, we selected 454 genes in the MEbrown module for further study.

### Functional annotation of RFs-related genes

To explore the molecular biological mechanisms of the RFs, GO and KEGG analyses of the RFs-related genes were performed. The BPs, CCs and MFs most related to RFs were ameboidal-type cell migration (**Figure 6C**), focal adhesion (**Figure 6D**), and growth factor binding (**Figure 6E**), respectively. Among signaling pathways, focal adhesion was identified as the most relevant (**Figure 6F**). The PPI network of the MEbrown module is depicted in **Figure 6G**, with KDR and CTNNB1 identified as hub genes. There were significant differences in the expression levels of KDR and CTNNB1 between the high- and low-grade groups and between the high- and low-risk groups. There were significant differences in the expression levels of KDR and CTNNB1 between the high- and low-grade and between the high- and low-risk groups (**Figures 6H–K**).

## Discussion

In this study, we performed subregional clustering within the tumor region on CECT images and constructed two ML models based on RFs from the tumor subregions to predict the pathological grade of ccRCC. These models demonstrated high predictive performance for the internal test set, with AUCs of 0.78 and 0.77. The consistency and robustness of this model were validated for an independent dataset, which showed similar performance, with AUCs of 0.74 and 0.77. These models can provide clinicians with a powerful tool for risk stratification before treatment and assist in customizing personalized treatment plans for patients. To enhance the interpretability and clinical utility of the models, we also explored the underlying potential biological significance of the RFs.

In radiomics research, different VOI segmentation strategies can lead to varying study outcomes. Traditional research methods often focus on segmenting the entire tumor region (11, 18–20), and some studies have considered the role of peritumoral components (21–24), however, none of these approaches can adequately reflect the internal heterogeneity of tumors. This study focused primarily on subregional radiomics, which can more precisely reflect the heterogeneity within tumors and offer multidimensional information. Existing studies have confirmed its value in survival prediction (25) and treatment response prediction (15). Our study demonstrated that models based on VOI_1_ offered superior performance in grading prediction. A possible explanation might be that VOI_1_, which represents low-density areas on CECT images, indicates regions of hypoxia, necrosis, or low cellular density within the tumor. These characteristics are typically closely associated with the aggressive behavior of the tumor and patient prognosis. Therefore, features extracted from VOI_1_ may provide more sensitive and specific biomarkers for tumor grading, which can improve prediction grading accuracy, tumor progression, and clinical outcomes.

Different phases of CT imaging offer unique insights into the diagnosis of ccRCC, but a consensus on the most effective phase has not yet been established. Luo et al. (21) reported that among four-phase CT images (unenhanced phase (UP), corticomedullary phase (CMP), nephrographic phase (NP), and excretory phase (EP)), features extracted from UP and EP images lead to better performance than features from other single/combined phase(s) do. Shu et al. (26) suggested that, compared with the CMP model, the NP and combined models were better at predicting the Fuhrman grade of ccRCC. In this study, we adopted parenchymal phase imaging for analysis. Compared with arterial phase images, parenchymal phase images display a more uniform distribution of contrast agent within the tumor, which aids in more clearly distinguishing between the tumor parenchyma and necrotic areas.

In the in-depth analysis of this study, we observed that the radscore was significantly greater in the groups with higher tumor grades and stages, suggesting a close association between the radscore and tumor aggressiveness and clinical severity. For the test set, the OS of patients in the high-risk group was significantly lower than that of patients in the low-risk group (*p* = 0.006), further demonstrating the potential of the radscore for predicting patient prognosis. However, for the training set, there was no significant difference in OS between the two groups (*p* = 0.058), which may be influenced by factors such as sample size and patient selection bias.

In our study, WGCNA was employed, and 454 RF-related genes were identified. Through GO and KEGG analyses, we found that these genes play crucial roles in key biological processes, such as cell migration, cell adhesion, and signal transduction, which are key contributors to tumor invasiveness and metastatic capacity (27–30). PPI network analysis further identified KDR and CTNNB1 as hub genes closely related to RFs. These genes exhibited significant expression variations across different risk subgroups, indicating their potential role in tumor development. KDR, a key regulator of angiogenesis, is highly expressed during tumor angiogenesis and is associated with poor prognosis. CTNNB1, a core component of the Wnt signaling pathway, is associated with the initiation and development of tumors when abnormally activated. The significant differences in the expression of these two genes suggest that they may serve as potential biomarkers and therapeutic targets. Our results indicate that RFs can be employed to predict the pathological grade of ccRCC, possibly because they are associated with genes and pathways related to cancer progression.

This study has several limitations. (1) The sample size of the study may limit the generalizability of the results, especially for the external validation set. (2) The CT scanners and scanning parameters used in the study could influence the extraction of radiomic features (RFs), and variations in equipment and parameters could potentially lead to feature variability. Additionally, differences in the timing of kidney contrast-enhanced scans across different centers may further contribute to this variability, potentially affecting the generalizability of our findings. (3) Although we employed subregional analysis to capture intratumor heterogeneity, it may not fully capture the complexity of all tumor subregions. (4) While associations between RFs and biological processes were explored, the direct biological significance and clinical relevance of these features still require further investigation. (5) Despite good performance with the internal test set and external validation set, the generalizability and long-term predictive accuracy of the models in broader clinical practice still need further validation. (6) This study focused on intratumoral features and did not incorporate peritumoral information, which could provide additional insights into tumor biology. Future studies should consider including peritumoral radiomic features to offer a more comprehensive assessment.

## Conclusion

In conclusion, we developed and validated two ML models based on subregional RFs for predicting the histological grade of ccRCC. These prediction models have great potential to guide clinical prognosis prediction and decision-making for therapy in the future. Furthermore, radiogenomic analysis revealed associations between RFs and pathways involved in cell migration, cell adhesion, and signal transduction, which are known to be related to the occurrence and development of tumors.

## Data Availability

The datasets presented in this study can be found in online repositories. The names of the repository/repositories and accession number(s) can be found in the article/**Supplementary Material**.

## Supplementary material

Supplementary Figure 1 Gaussian mixture model (GMM) clustering of radiomics feature using principal component analysis (PCA)-reduced data. **(A–I)** the clustering results for 2 to 10 components, respectively. Points represent samples, colored by cluster. Red stars indicate cluster centroids.

Supplementary Figure 2 Association between radscore and clinical characteristics of ccRCC. **(A)** The landscape of radscore-related clinical characteristics of ccRCC in the external validation set. **(B–E)** Differences in radscore between different clinical subgroups in the external validation set.

Supplementary Figure 3 Waterfall plot showing the distribution of Radscore between high-grade and low-grade groups. **(A)** training set. **(B)** Internal test set. **(C)** External validation set.
