# Supplementary figures and images for: Subregion-based radiomics analysis for predicting the histological grade of clear cell renal cell carcinoma
Source: Front Oncol. 2025 May 27;15:1554830. doi: 10.3389/fonc.2025.1554830 (PMC12149422; doi:10.3389/fonc.2025.1554830)

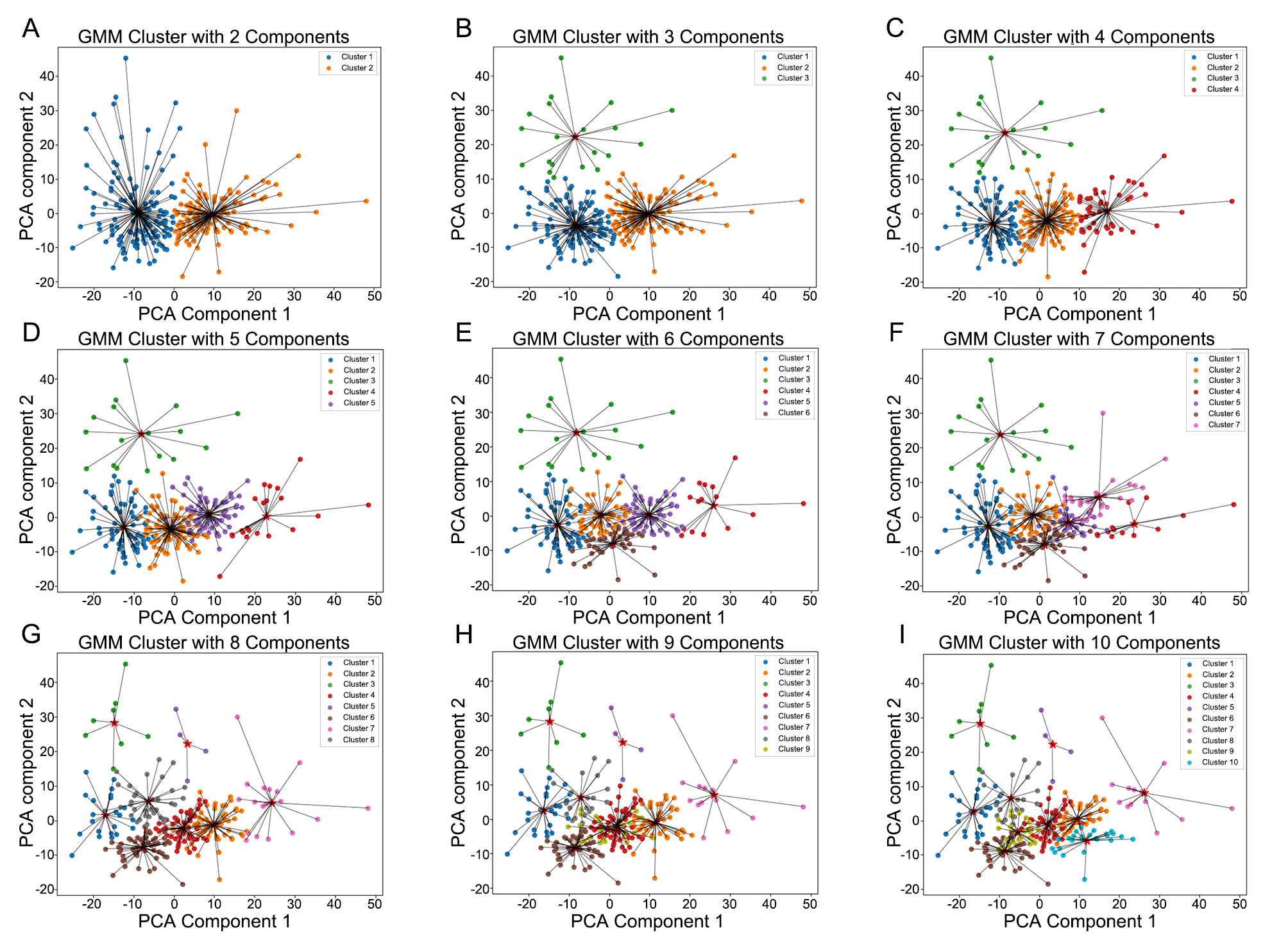

Supplement: Supplementary Figure 1 — Gaussian mixture model (GMM) clustering of radiomics feature using principal component analysis (PCA)-reduced data. (A–I) the clustering results for 2 to 10 components, respectively. Points represent samples, colored by cluster. Red stars indicate cluster centroids. [file Image1.tif]

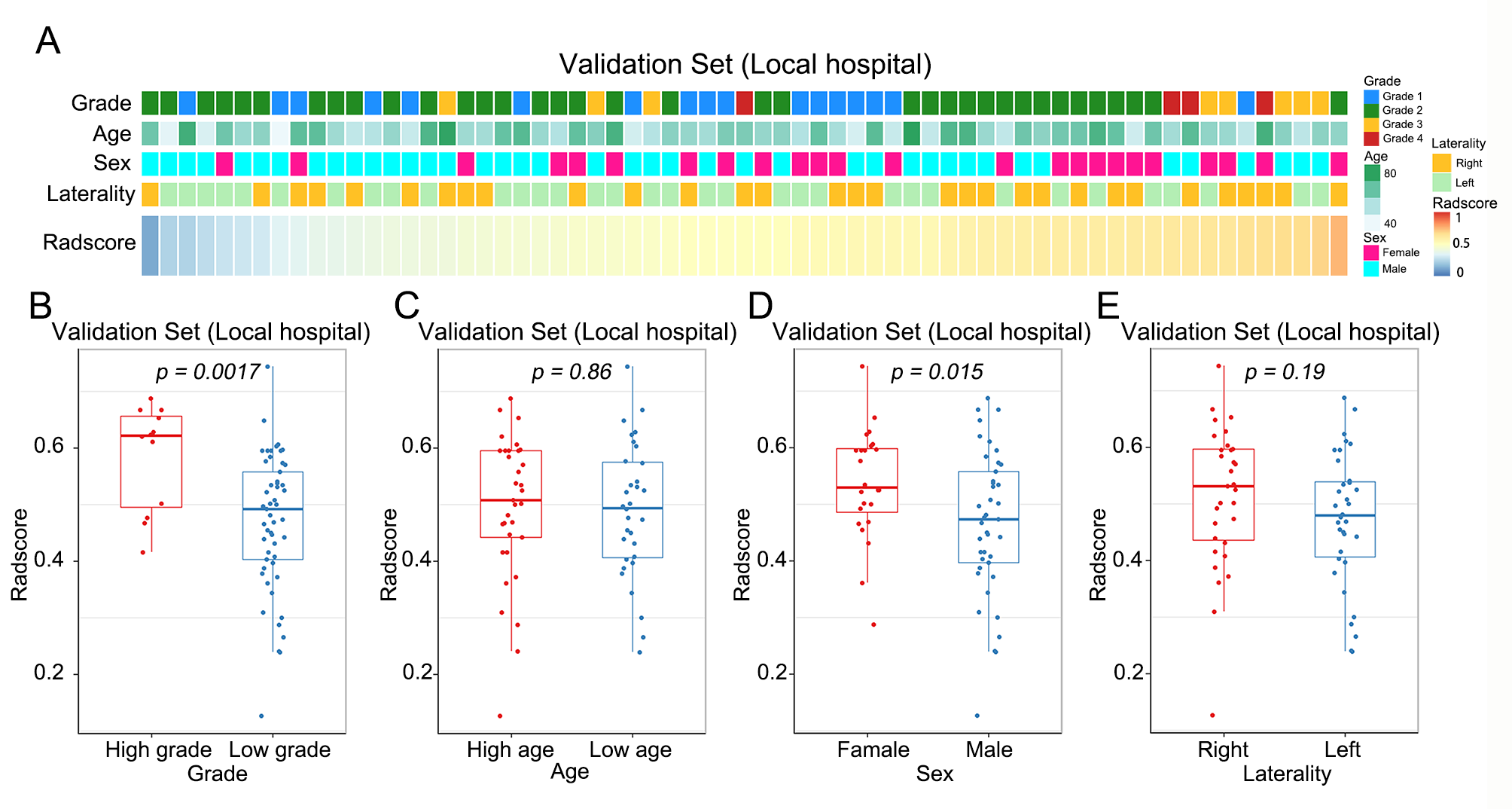

Supplement: Supplementary Figure 2 — Association between radscore and clinical characteristics of ccRCC. (A) The landscape of radscore-related clinical characteristics of ccRCC in the external validation set. (B–E) Differences in radscore between different clinical subgroups in the external validation set. [file Image2.tif]

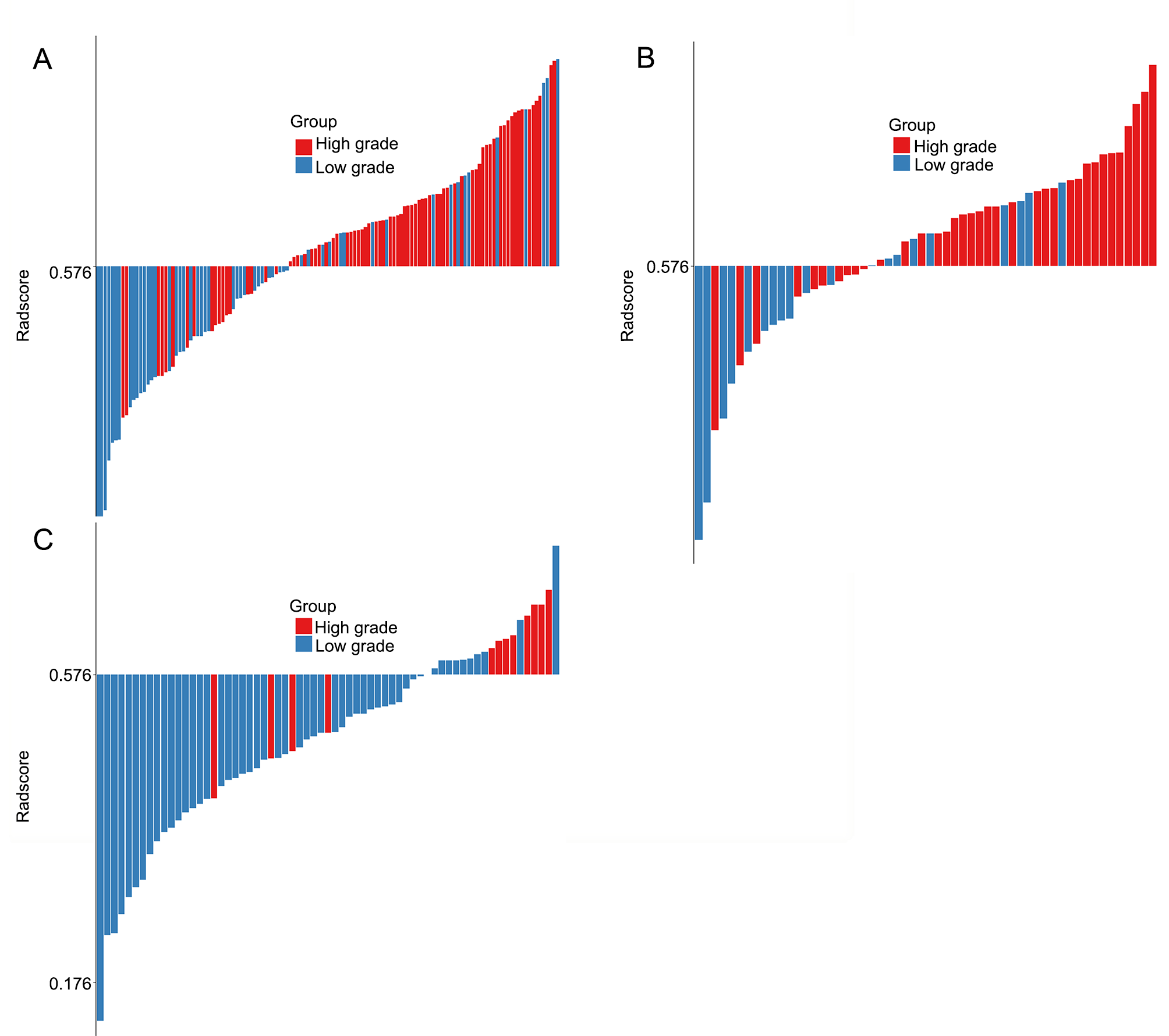

Supplement: Supplementary Figure 3 — Waterfall plot showing the distribution of Radscore between high-grade and low-grade groups. (A) training set. (B) Internal test set. (C) External validation set. [file Image3.tif]
