# Supplementary material for: Subregion-based radiomics analysis for predicting the histological grade of clear cell renal cell carcinoma
Source: Front Oncol. 2025 May 27;15:1554830. doi: 10.3389/fonc.2025.1554830 (PMC12149422; doi:10.3389/fonc.2025.1554830)
Supplement: Supplementary file 5 [file DataSheet1.docx]

The extracted features were grouped into the following four categories: Category 1, first-order statistics, n = 18; Category 2, shape and size features, n = 14; Category 3, textural features, n = 75, including grey-level cooccurrence matrix (GLCM), n = 24, grey-level run length matrix (GLRLM), n = 16, grey-level size zone matrix (GLSZM), n = 16, grey-level dependence matrix (GLDM), n = 14, and neighbouring grey tone difference matrix (NGTDM), n = 5; and Category 4, filter-derived features, filter ‘wavelet’, n = 744, filter ‘LoG’, n = 279.
