# Supplementary material for: Subregion-based radiomics analysis for predicting the histological grade of clear cell renal cell carcinoma
Source: Front Oncol. 2025 May 27;15:1554830. doi: 10.3389/fonc.2025.1554830 (PMC12149422; doi:10.3389/fonc.2025.1554830)
Supplement: Supplementary file 6 [file DataSheet2.docx]

Radscore = b + a1X1 + a2X2 + a3X3+......+aiXi, where Xi represents the selected RF, ai is the regression coefficient of the corresponding RF, and b is the intercept. The calculation formula of the radscore was as follows:

Radscore = 0.584615+log-sigma-2-mm-3D_glcm_Idn x 0.074830+log-sigma-3-mm-3D_glcm_Idmn x 0.002750-wavelet-LHL_glcm_Correlation x 0.003898
